# Study Protocol for a Randomized Double Blind, Treatment Control Trial Comparing the Efficacy of a Micronutrient Formula to a Single Vitamin Supplement in the Treatment of Premenstrual Syndrome

**DOI:** 10.3390/medicines3040032

**Published:** 2016-12-07

**Authors:** Hannah Retallick-Brown, Julia Rucklidge, Neville Blampied

**Affiliations:** Department of Psychology, College of Science, University of Canterbury, Private Bag 4800, Christchurch 8041, New Zealand; Julia.rucklidge@canterbury.ac.nz (J.R.); Neville.blampied@canterbury.ac.nz (N.B.)

**Keywords:** premenstrual syndrome, premenstrual dysphoric disorder, treatment, vitamins, minerals

## Abstract

**Background:** The recent addition of Premenstrual Dysphoric Disorder (PMDD) to the Diagnostic and Statistical Manual (5th ed.) has highlighted the seriousness of this disorder. Many alternatives to psychoactive medication in the form of vitamins, minerals, and plant extracts have been trialled by women seeking a natural treatment approach. We plan to explore whether a well validated micronutrient formula, EMPowerplus Advanced, can outperform a recognized single nutrient treatment, vitamin B6, for the treatment of Premenstrual Syndrome (PMS). **Methods:** This will be a randomized treatment control study. Eighty women will be recruited and assigned to one of two treatment groups; EMPowerplus Advanced or vitamin B6. Baseline daily data will be collected for an initial two cycles, followed by three months of active treatment. A natural follow up will take place three cycles post treatment. **Results:** The primary outcome measure will be PMS change scores as based on results from the Daily Record of Severity of Problems (DRSP). The number of treatment responders for each of the two groups will yield a comparison score between the two treatments, with participants deemed as a responder if they show a total PMS score improvement of 50% from their baseline scores on the DRSP. **Conclusion:** If a micronutrient formula proves more effective for treating PMS, not only does it give women suffering from the condition a viable treatment option, but it may also suggest one cause of PMS; that is insufficient minerals and vitamins.

## 1. Introduction

The average woman will experience 13 menstrual cycles per year, corresponding to roughly 451 cycles over her lifetime and nearly 35 years of menstruation [1]. As many as 90% [2] of women will experience mild physical or mental symptoms in the week leading up to menstruation, and for some the symptoms are severe. Approximately 20% to 40% of ovulating women will suffer from numerous symptoms, collectively termed; premenstrual syndrome (PMS) [3]. An even more symptomatic form of PMS, known as Premenstrual Dysphoric Disorder (PMDD) is found in 1.8%–5.8% of women [4]. For women meeting criteria for either PMS or PMDD the symptoms include: affective lability, irritability, anger, increased interpersonal conflict, depressed mood, anxiety, difficulty concentrating, fatigue, change in appetite, sleep disturbance, feelings of being out of control, less interest in normal activities as well as physical symptoms such as bloating, breast swelling, and joint pain [4]. The hormonal induced changes affect all women regardless of ethnicity or socio-economic status and place a burden on interpersonal relationships, mental health, and work productivity [3].

The American College of Obstetricians and Gynecologists (ACOG) recommend pharmacological treatment should be used as a first line intervention for PMDD [5]. Selective serotonin reuptake inhibitors (SSRIs) have demonstrated a small effect size in PMS change scores (standardized mean difference= −0.36, 95% CI −0.2 to −0.51) [6], although they are more effective in women with severe PMS/PMDD; showing a moderate effect size in overall symptom reduction in this population of women (standardized mean difference= −0.53, 95% CI 0.68 to −0.39) [7]. However, the use of anti-depressants in the treatment of PMS can, at times, cause significant side effects including nausea, loss of libido, fatigue, insomnia, dry mouth, gastrointestinal complaints, and tremors (among other problems) [8], which can cause many women to terminate treatment. Women are far more likely to terminate participation when in an active treatment group compared to women on placebo, with higher frequencies of side effects observed in the former group. However, results from SSRI trials have produced incomplete side effect results, with many studies neglecting to specify side effects [8]. The second line of pharmacological treatment, hormone replacement therapies, interrupt the process of sex hormone production, causing anovulation. Due to serious side effects including reduction in mineral bone density, induction of postmenopausal symptoms and induction of symptoms similar to those seen in PMS such as headaches, lowered mood, and muscle tenderness [9,10], gonadotropin-releasing hormones (GnRH) agonists are used sparingly. Meanwhile, oral contraceptives have shown poor treatment efficacy and are not a recommended treatment [11]. Women with less severe symptoms are advised to try lifestyle changes such as dietary alterations, exercising regularly, improving sleep length and quality, and alleviating stress [12]. As current frontline medical treatments for PMS can have significant side effects, there is a need for new forms of treatment.

Vitamins and minerals have a long history of being studied for the treatment of mental illness, usually one nutrient at a time [13]. A lack of specific vitamins and minerals (micronutrients) has been thought to be a causal factor in PMS and therefore their supplementation provides a possible treatment avenue [14]. Vitamin B6 (pyridoxine) has received much attention for its use in alleviating premenstrual symptoms. It has been hypothesized that B6 works, at least in part, through synthesizing serotonin, a neurotransmitter implicated in depression [15]. Numerous randomized controlled trials (RCTs) have studied the vitamin, finding promising results. For example, in a randomized crossover trial Doll, et al. [16] found pyridoxine to be effective in a dose as little as 50 mg per day on the emotional symptoms of depression and irritability and on tiredness. Kashanian, et al. [17] in a placebo controlled trial showed that at 80 mg per day pyridoxine exerted a significantly stronger effect on psychiatric symptoms compared to placebo, while both significantly reduced somatic symptoms. Wyatt, et al. [18] carried out a systematic review of the literature involving vitamin B6 and its use in the treatment of premenstrual syndrome. They noted that a number of studies were of poor quality but they did find evidence in support of the use of vitamin B6. In particular, the odds ratio for active treatment over placebo in alleviating overall premenstrual symptoms was 2.32 (95% confidence interval 1.95 to 2.54).

While a number of other nutrients including calcium, vitamin E, magnesium have shown some evidence of efficacy in treating PMS [15,19], the overall results are generally modest, suggesting that the one disease one treatment model fails to take in the complexities of human nutrient requirements [20]. 1200 mg of calcium a day over three menstrual cycles produced a 48% reduction in PMS symptoms compared to 30% in the placebo group [21]. Vitamin E has been indicated as a possible treatment, yet results have failed to reach significance [22]. Magnesium has shown variable results in treating the symptoms of PMS [23]. When combined with 50 mg of vitamin B6, magnesium at a dose of 200 mg/day was effective on multiple PMS symptoms despite a relatively short treatment duration of one month [24].

Given that micronutrients are implicated in a range of essential brain functions from neurotransmitter synthesis to powering brain metabolism [25] and work synergistically, there is a growing body of literature exploring a much greater combined approach. Indeed, broad spectrum micronutrient formulas have been found to be effective in treating a multitude of symptoms typically seen in PMS such as irritability, low mood, poor sleep and anger [26]. There is a growing body of literature demonstrating the benefit of using broad spectrum micronutrients in the treatment of a variety of psychological/psychiatric conditions such as anxiety, depression, bipolar, attention-deficit/hyperactivity disorder, and obsessive compulsive disorder in both adults and children and this approach is proving to be far more successful than any single nutrient alone [26]. Previous research has already explored the use of a multi-ingredient formula in the treatment of PMS [27,28,29]. However, the formula, “Optivite”, contained high doses of both magnesium (250 mg/day) and vitamin B6 (300 mg/day) when taken at the full recommended dosage. Single nutrient doses as high as those seen in Optivite can be considered a standalone treatment in their own right, and indeed have been used in other studies [18,23,24]. Therefore, the results have been somewhat obscured by the high single nutrient dosages; was it the multi-ingredients or the vitamin B6/magnesium alone that resulted in improved PMS symptoms? The current study intends to clarify the efficacy of a broad spectrum formula in the treatment of PMS, using doses of single nutrients below the therapeutic levels of single nutrient treatments reported in previous studies.

The most studied broad spectrum micronutrient formula is called EMPowerplus (EMP+) which consist of 36 ingredients, the majority of which are minerals and vitamins (although it is sold variously as EMPowerplus Advanced, Daily Essential Nutrients and Q96). EMP+ has already shown efficacy in treating similar symptoms to those seen in the premenstrual phase such as low mood, anxiety, rage, insomnia and loss of control [26]. Given its efficacy in assisting with such symptoms, it is hypothesized that EMP+ will prove efficacious in women suffering from premenstrual complaints. This paper presents the study protocol for the Natural Treatment of Premenstrual syndrome (NTP) trial. This is a randomized treatment controlled trial that aims to investigate the effectiveness of micronutrients in the treatment of premenstrual syndrome (PMS). We hypothesize that women receiving EMP+ will experience greater improvements in PMS symptoms than women receiving vitamin B6.

## 2. Methods

### 2.1. Study Design

This study will follow women for a total of eight menstrual cycles; two cycles of pretreatment, three cycles of active treatment, and a three cycle natural follow up. It is a randomized, parallel group, double blind, treatment controlled trial of natural supplement interventions for moderate to severe premenstrual syndrome (PMS). We are currently enrolling 80 women from the greater Canterbury region (New Zealand). Recruitment and intervention are anticipated to occur over a 1.5–2 year time frame. Participants will be randomized to receive either a micronutrient formula, EMPowerplus Advanced (EMP+), or an active comparator treatment, vitamin B6.

### 2.2. Study Aims

We aim to investigate the efficacy of a micronutrient supplement (EMP+) in the treatment of PMS as measured by the Daily Record of Severity of Problems (DRSP) questionnaire. We are particularly interested in comparing EMP+ to an already efficacious single-ingredient natural treatment, vitamin B6. We hypothesize that those participants receiving EMP+ will have greater improvement not only in their premenstrual symptoms but also on secondary measures of anxiety, depression, stress, sleep, sexual functioning, and quality of life. As well as looking at premenstrual symptoms change, we will record the number of treatment responders for each of the two treatments and compare the different rates of response. A participant will be deemed to be a responder if they show a total PMS improvement score of 50% or more as based on baseline scores from the DRSP. A further aim is to identify any mediation or moderation effects of stress sensitivity on PMS ratings.

### 2.3. Participant Eligibility

All participants must be over 18 years of age, experience regular menstrual cycles, must not be pregnant or breast feeding or attempting to become pregnant, and currently cannot be taking other medications for the treatment of PMS. If participants are using sex hormones other than the contraceptive pill they will also be excluded (these include gonadotropin-releasing hormone agonists, anti-estrogens, androgens, and estrogens specifically targeting PMS symptoms); however, as long as their contraceptive pill dosage stays the same throughout the study they are eligible to participate. Any dose or contraceptive pill change will be considered a protocol violation and the participant will therefore count as a drop out and will not be included in the per-protocol analyses. Final exclusion criteria include the presence of a current mood disorder (other than PMDD), a neurological disorder involving brain or other central nervous system function (e.g., epilepsy), evidence of untreated or unstable thyroid disease, any known abnormality of mineral metabolism (e.g., Wilson’s disease, hemochromatosis), judged clinically to be at serious risk of suicide or violence, or currently taking any other medication with primarily central nervous system activity.

### 2.4. Sample Recruitment

Community based recruitment strategies are to be employed to recruit participants. Methods of recruitment include posters around Christchurch, at public health centers, and at the University of Canterbury, as well as advertising on social media outlets, namely Facebook.

Prospective participants will complete an initial screening questionnaire via the study website (http://bit.ly/nutritionandPMS). If the participant meets the preliminary inclusion criteria they are invited to attend further face-to-face screening at the university based laboratory.

### 2.5. Treatment

Participants will be randomized to receive either vitamin B6 or EMP+, the full list of ingredients and doses for both formulas can be viewed in Table 1. Numerous studies have used EMP+ for the treatment of mental health concerns, with no occurrences of significant adverse outcomes observed [30]. Vitamin B6, at very high doses, 2000 mg/day has been shown to cause peripheral neuropathy (Joint Food Safety and Standards Group, 1997). However, the same, but reversible effects have been noted in participants taking 200 mg/day [31]. Therefore, the current study is using 80 mg/day, a dose that has proven effective on both physical and emotional symptoms of PMS without co-occurring side effects [17].

### 2.6. Study Procedure

#### 2.6.1. Screening Assessment

Prospective participants will complete a modified version of the Daily Record of Severity of Problems (DRSP) online to confirm the presence of menstrual related symptoms in the luteal phase of their menstrual cycle. They must meet the following to be included: (1) during the mid-follicular phase (day 6–10 after the onset of menses), their symptoms must be no more than “mild”; (2) in the week prior to menses, at least one emotional symptom i.e., depression, anxiety, affective liability, or anger must be “moderate” or higher; (3) during this phase they must also show a worsening in at least three out of the eleven symptoms covered in the DRSP questionnaire; (4) and finally these symptoms must have a “moderate” impact on their quality of life, as indicated by the participant’s endorsement of one of three questions in the DRSP. These initial screening criteria are again to be confirmed during the double cycle baseline period. Judgments of “mild”, “moderate” and so on will be based on the numerical value the participant ascribes to her symptom, where; 1 = not at all, 2 = minimal, 3 = mild, 4 = moderate, 5 = severe, 6 = extreme.

**Figure 1:**
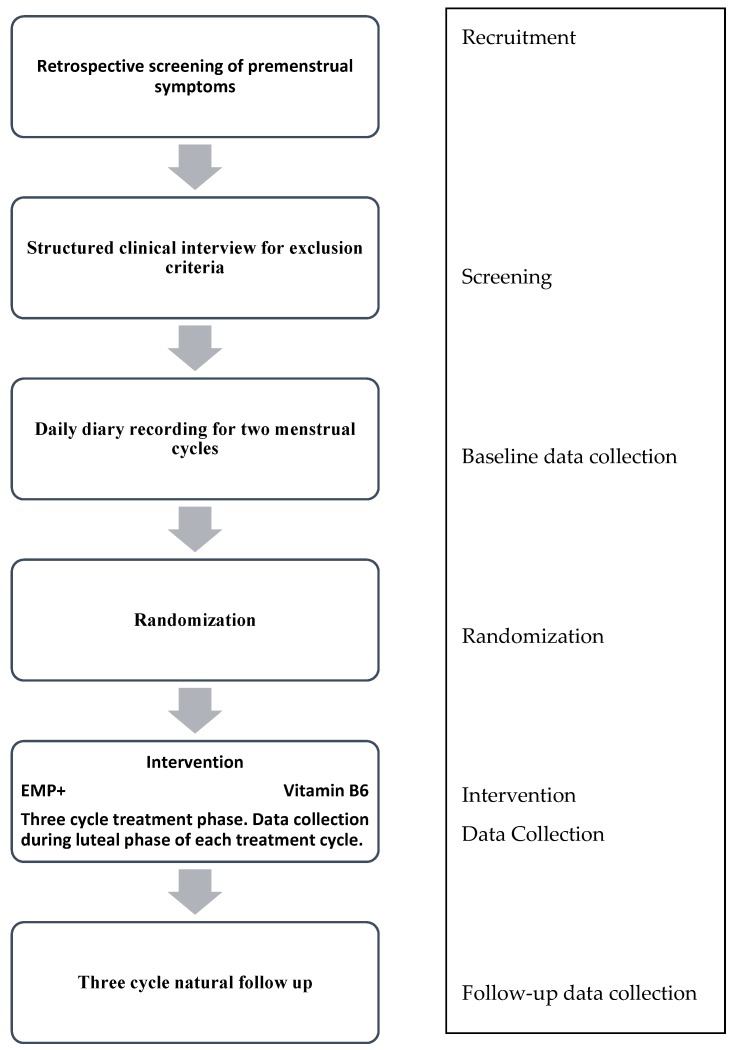
Study Process.

Women who meet the above criteria will be invited to attend a meeting where current and past mood disorders are assessed using a psychiatric diagnostic interview (Structured Clinical Interview for DSM-5, Research Version) [32]. Any participants who are deemed to have a current mood disorder, other than Premenstrual Dysphoric Disorder (PMDD), will be excluded at this point. In instances where mood disorders are detected the clinical psychologist overseeing the study will be notified and referrals will be made to appropriate services, as deemed necessary by the psychologist. Likewise, any concerns arising during the baseline or treatment phase will be managed in the same manner; however, were a participant to develop a mood disorder during the treatment phase they would not be excluded from the study at this point. For an overview of the study process, please refer to Figure 1.

#### 2.6.2. Randomization and Allocation

If an individual meets inclusion criteria, consents to participate in the study, and completes the two cycles of baseline, they will then be allocated the next available randomized participant number from a previously generated list. Participants are to be randomized in a 1:1 ratio to the micronutrients or vitamin B6 condition by a research assistant using block randomization [33]. The pharmacist will receive this randomization list. They will then package all capsules (micronutrient and vitamin B6) in plain white containers, labelled simply with the participant number and daily dose requirements. A sealed envelope containing the treatment allocation for each participant will be kept in a secure location, only to be opened in the case of an emergency (e.g., a serious deterioration in the participant’s health), meaning the blind will be broken for that participant only. A separate randomization sheet is kept by the research assistant and is to be given to the statistician for analysis of the primary outcome at the end of data collection, and the researcher for analysis of the secondary outcomes.

#### 2.6.3. Blinding

Both participants and researchers will be blind to the treatment condition of each individual. Randomization will be conducted by a research assistant who will not be involved in any other aspect of the study. There is no difference between the appearance of the two supplements, and both contain riboflavin to ensure the change in urine color this vitamin causes is universal across the conditions. The dose of the vitamin B6 has been distributed across 8 pills such that the group randomized to vitamin B6 consumes the same number of pills. In order to fill the capsules, other inert ingredients are being given alongside vitamin B6 including; acacia gum, cocoa powder, maltodextrin, and steric acid, none of which are derived from wheat so the formula is safe for coeliacs to consume. Once all data are entered and analysis of the primary outcome measure is complete, the researchers will complete analysis of the secondary outcomes using a randomization list labelled A or B. Once all analyses are complete the blind will be broken and participants will be informed of their treatment allocation.

#### 2.6.4. Intervention

Capsule dose will be titrated up over six days to the full dose of eight capsules/day (two doses of four capsules taken with water and food). Participants will continue to take the capsules for a full three menstrual cycles, as women with a cycle that lasts between 21–35 days are eligible for this study, the amount of time a participant is on active treatment will vary as a function of their cycle length. Based on previous clinical trials, participants usually describe a gradual effect of micronutrient supplements that reaches full effect within four weeks, therefore the three cycle treatment phase should, theoretically, afford enough time for treatment to reach its full effect.

During each of the three treatment cycles, participants will come into the University during the end of their PMS phase to complete questionnaires, receive new capsules and return old capsules and their DRSP diary. For each visit made to the University, following the initial baseline meeting, participants will receive a NZD 10 voucher able to be used at all major petrol stations across New Zealand. Treatment compliance is to be measured via counting and recording capsule consumption, whereby less than 80% consumption of the assigned dose will be considered non-compliance. Participants will return their remaining capsules at the end of the treatment phase in order for compliance to be assessed.

At the end of the treatment phase, participants will decide on their own course of treatment; taking vitamin B6, EMP+ or another active treatment of their choosing or discontinuing treatment altogether (they will remain blind to the treatment they were receiving). No treatment will be supplied by the study once participants have completed their three cycles of treatment, however, information on how to obtain vitamin B6 or EMP+ will be provided. Three cycles later participants will be contacted via email to complete a modified version of the DRSP online, as well as answer questions about their current PMS management, including treatment use over the preceding three cycles. The modified version of the DRSP will ask participants to rate their symptoms as usual on the 6 point scale; however, instead of completing the diary for each day of their PMS phase they will instead give an overall rating for each symptom at the end. Three cycles without treatment, if the participant chooses to do so, should ensure enough time to see a return of pre-treatment PMS scores should that happen.

### 2.7. Data Collection and Outcome Measures

Table 2 displays the outcome measures and time points at which they are to be collected. Demographic data including premenstrual syndrome history and previous and current mental health status is to be collected in the initial screening questionnaire. Self-report measures are obtained from each participant at baseline, and during each cycle of treatment to determine depressive, anxious, and stress symptoms, sleep quality, sexual satisfaction, and quality of life as well as general information on food and drug and alcohol intake.

#### 2.7.1. Primary Outcome Measure

During the participant’s first two menstrual cycles, daily data on premenstrual symptoms is to be collected via the *Daily Record of Severity of Problems (DRSP)* questionnaire [34]. The DRSP is a commonly used screening tool for premenstrual dysphoric disorder [2]. It provides a severity rating from 1 (not at all) to 6 (extreme) for 11 questions assessing premenstrual symptoms and asks a further three that look at the impact the symptoms have in terms of productivity, participation in normal activities, and relationship harmony [34]. For each cycle of treatment, participants will fill out the DRSP for the 10 days that correspond with their PMS phase. At natural follow up women will complete a modified version on the DRSP that will ask about their symptoms over the preceding week (again at the point in their cycle that corresponds with their PMS phase).

#### 2.7.2. Secondary Outcome Measures

*Third party symptom ratings:* Participants, who agree, will be asked to provide details of a third-party observer who is able and willing to comment on the participant’s PMS symptoms. Observers will do this via an online, modified version of the DRSP questionnaire that is to be filled out twice in the luteal phase; once at baseline and again at the end of the treatment.

Each cycle during the treatment phase and once at baseline, participants will fill out the following questionnaires; Depression and Anxiety Stress Scales-42 (DASS-42), Women’s Quality of Life Questionnaire (WOMQOL), Pittsburgh Sleep Quality Index (PSQI), The Perceived Stress Scale (PSS), The New Sexual Satisfaction Scale-Short form (NSSS-S), a side effects questionnaire, and a drug, alcohol, and food questionnaire (DAF).

The DASS-42 is a 42 item self-report instrument designed to measure three related negative emotional states; depression, anxiety, and tension/stress [35]. It is used across a variety of clinical studies and as women with PMS/PMDD commonly experience symptoms measured by the DASS-42 it provides an additional useful tool for tracking psychometric change.

Contextual, common every day stress will be measured by the PSS 10-question version [36]. Participants are asked to rate the stressfulness of a number of events from the past week of their life in a modified version of the PSS. While there are three versions of the PSS, the current study will use the 10-question version which has been well validated. Questions are rated on a 0–4 point scale, focusing on relatively general stressors that pertain to every day events as opposed to large-scale incidents such as death or environmental disaster [36]. The PSS was found to be a good predictor of both health and health-related outcomes, and while it is correlated with depressive symptomatology it was found to measure an independent construct. In a sample of college students the PSS was found to have good test-retest reliability (*r* = 0.85), an important factor for this study.

The WOMQOL was specifically designed to assess a woman’s quality of life in four domains: physical, psychological, social, and spiritual wellbeing [37]. Forty questions have three possible answers; Yes, No, or Not Applicable with higher overall scores indicating a better quality of life. Quality of Life scales are frequently used in chronic illness and disease studies as a measure of disease impact and treatment response [3].

The New Sexual Satisfaction Scale-Short Form (NSSS-S) is comprised of 12 items which are measured on a 5-point scale [38]. Two factors are covered in this questionnaire; personal experiences and sensations, and partner’s general behaviors and sexual activity, with higher scores indicating greater sexual satisfaction [38]. The form shall be modified slightly in time period; instead of asking about sexual satisfaction over the last six months, participants will assess their satisfaction over the last *week*. Only those women who are currently sexually active (excluding solo sexual activity) will be asked to fill out the questionnaire.

Pittsburgh Sleep Quality Index (PSQI) measures sleep quality and disturbances over a one month period [39]. There are 19 questions which yield seven different measures; subjective sleep quality, sleep latency, sleep duration, habitual sleep efficiency, sleep disturbances, use of sleeping medication, and daytime dysfunction. These scores can then sum together to give an overall sleep quality score. The PSQI has been found to have sound reliability and validity [39].

Drug, Alcohol, and Food Questionnaire (DAF). Questions regarding dietary patterns over the previous week will be assessed using a questionnaire modified from Baker, et al. [40]. In this study, a healthy eater will be defined as someone who eats in a balanced way, eats three meals a day, doesn’t eat too much junk food, eats moderate amounts, and stops eating when full. Participants are also asked to indicate from 1 (<once a week) to 5 (daily) how often over the previous 2 weeks they ate breakfast, ate a balanced meal, ate even when full, ate lots of fruits and vegetables, and ate fast foods or snack foods such as potato chips or candy bars. Three items are reverse scored. They will also be asked about average daily servings of fruit and vegetables (from 1 (<one serving) to 5 (4 or more servings)), and to indicate from 1 (not very healthy) to 7 (very healthy) how healthy they think their diet is. Total scores will range from 9 to 47, with a higher score indicative of a healthier diet. These specific questions were recently adapted and validated by Kuijer and Boyce [41]. In the validation study, Kuijer and Boyce showed that the questions were correlated with a 2 week diary report of those behaviors. Moreover, the retrospective recall was found to be a fairly accurate estimate of the eating behaviors as reported during the diary period. The summing of the items has been used successfully in other studies such that a higher score on the summed scale indicates healthier eating behaviors (Cronbach’s alpha 0.67) [41]. In the course of answering questions on food intake, participants will also be asked about alcohol and drug consumption over the preceding week.

A scale assessing common side effects for both treatment groups will be administered throughout the study, including baseline. Participants will be asked to indicate whether any symptoms commonly identified as side effects were experienced during treatment and then asked what actions they have taken to remedy them.

In the final set of questionnaires, at the end of the third cycle of intervention, participants will be asked to complete the Clinical Global Impression scale (CGI scale) [42]. The CGI scale is used in numerous clinical studies to evaluate the global efficacy and safety of a medication [43]. Participants will judge their own improvement at the end of the trial on the Clinical Global Impression-Improvement scale (CGI-I) in a self-rated version rather than a clinician rated scale.

### 2.8. Data Management

All study data is to be contained in locked storage systems; either a password protected computer system at the University of Canterbury or on a web-based data collection system (www.canterbury.qualtrics.com) for electronic documents, while hard copies will be kept in secure filing cabinets at the university.

### 2.9. Study Integrity

The trial prospectively registered under the Australian and New Zealand Clinical Trials Registry (ANZCTR). Trial Identification = ACTRN12615000131550.

Universal Trial Number (UTN) = U1111-1164-2407.

Ethics approval was granted through the University of Canterbury Human Ethics Committee: HEC 2014/129, on 13 November 2014. Approval was updated on 4 February 2015 following trial amendments. Written informed consent is to be granted by all participants before entry into this study.

### 2.10. Sample Size

Determining the sample size was a challenge based on the diversity of studies that have thus far been conducted. We are aiming to recruit 40 participants per group, making a total of 80 participants. The final sample size was calculated via a sample size calculation where the best estimate of the effect size (ES) of vitamin B6 on psychiatric symptoms was 0.36 [17], while EMP+ produced (in a conservative estimate) an ES of 0.64 on mood symptoms in a blinded trial [44]. Yet in an open label trial the reported effect size was 1.96 [45]. To detect a medium ES of 0.6 in this study, a total of 72 participants are needed. This number has been increased to 80, 40 per-group, to allow for attrition. We have chosen to detect a medium effect as we believe this will be clinically relevant for those women choosing to use natural supplements in treating PMS. Based on the available data is it reasonable to expect to see a medium ES difference between the two treatments; currently estimates for mood in blinded micronutrient trials appears to be medium to large [44].

### 2.11. Data Analyses

The first participant enrolled in this study on the 1 March 2014, final data collection is expected to be completed by May 2017.

Descriptive statistics including age, cycle length, menstruation length, duration of premenstrual complaints and occupation, will be documented in order to compare the two treatment group’s homogeneity to each other and to the wider population. Two-tailed *t*-tests or ANOVA will be used to identify any group differences.

The primary efficacy analysis will assess average treatment group differences, as well as individual differences for the primary outcome measure (DRSP) using a repeated measures design. Repeated-measures ANCOVA will be used to assess changes from baseline to end of treatment between both treatment groups, using the baseline level as the covariate. Time series graphs will also be constructed to show the intensity and trajectory of change in symptom patterns overtime at both an individual and treatment group level. Finally, modified Brinley Plots [46] will be constructed to show overall change, along with Reliable Change for each individual, overall %Reliable Positive Change, and Cohen’s d Effect Size. Pre and post treatment data points for each participant will be placed onto a scatter plot, where the direction of desired change and clinical cut-offs are indicated to assist interpretation. Separate plots will be drawn for each secondary measure of sleep, stress, sexual satisfaction, quality of life, and symptoms of depression, anxiety and stress.

Intention to treat (ITT) analysis will be employed, thus all participants will be included regardless of whether they complied with capsule consumption. Treatment adherence will be based on total treatment consumption; less than 80% will be considered non-treatment adherence. Per-protocol analysis will be performed as secondary analysis of DRSP change, whereby participants with protocol violations (drop-out, incomplete treatment consumption) will be excluded. Those participants who drop out will be classified based on treatment group and reason for discontinuation. Categorical outcome of drop out will then be compared between the two treatment groups using Chi-square tests with a 95% confidence interval and Odds Ratios.

Hierarchical linear regression will be used to investigate baseline stress sensitivity as a possible mediator/moderator of treatment outcome. Effect sizes will be calculated using Cohen’s d guidelines, while an alpha level of 0.05 will be set on all tests of treatment effects.

## 3. Discussion

Many women seek a natural solution to treating their monthly premenstrual symptoms. Single ingredient treatments have shown some promise in improving both emotional and physical symptoms of PMS [11], yet there is a gap in our knowledge about the efficacy of combined micronutrients. Previous research in this area has focused on multi-ingredient formulas high in specific vitamins, clouding the effects of a broad spectrum treatment [27]. The current study aims to assess the efficacy of a broad spectrum micronutrient formula, and compare it to an already established natural treatment; answering in the process the question of “is a multi-ingredient formula better than a single vitamin treatment for PMS?” Additionally, a secondary aim is to assess and compare the wide ranging effect of both treatments on sleep, sexual satisfaction, quality of life, stress, anxiety, and depression. Stress sensitivity, conceptualized as an individual’s baseline stress level and their response to stress, is to be further investigated for its possible moderating/mediating effect on PMS symptoms.

## Figures and Tables

**Table 1:** Nutrient Information.

**EMPowerPlus Advanced Supplement Facts**			
Amount Per Serving (8 capsules)			
Vitamin A (as retinyl palmitate)	3072 IU	Calcium (as chelate)	704 mg
Vitamin C (as ascorbic acid)	320 mg	Iron (as chelate)	7.328 mg
Vitamin D (as cholecalciferol)	768 IU	Phosphorus (as chelate)	448 mg
Vitamin E (as d-alpha tocopheryl succinate)	192 IU	Iodine (from Pacific kelp)	108.8 mcg
Thiamin (as thiamin mononitrate)	9.6 mg	Magnesium (as chelate)	320 mg
Riboflavin	7.2 mg	Zinc (as chelate)	25.6 mg
Niacin (as niacinamide)	48 mg	Selenium (as chelate)	108.8 mcg
Vitamin B6 (as pyridoxine hydrochloride)	19.2 mg	Copper (as chelate)	3.84 mg
Folic acid	768 mcg	Manganese (as chelate)	5.12 mg
Vitamin B12 (as methylcobalamin)	480 mcg	Chromium (as chelate)	332.8 mcg
Biotin	576 mcg	Molybdenum (as chelate)	76.8 mcg
Pantothenic acid (as calcium pantothenate)	11.52 mg	Potassium (as chelate)	128 mg
Propriety blend: Choline bitartrate, dl-phenylalanine, citrus Bioflavonoids, Inositol, l-Glutamine, l-Methionine, Grape seed extract, Gingko biloba leaf extract, germanium sesquioxide, Boron (as chelate), Vanadium (as chelate), Nickel (as chelate). Other ingredients: capsule shell (gelatin, titanium dioxide) microcrystalline cellulose, glycine, citric acid, magnesium stearate, silicon dioxide, mineral wax			
**Vitamin B6 Supplement Facts**			
Amount Per Serving (8 capsules)			
Vitamin B6 (as Pyridoxcine HCI)	80 mg	Riboflavin	0.8 mg
Acacia Gum	2400 mg	Cocoa Powder	3167.2 mg
Other ingredients: maltodextrin, and steric acid			

**Table 2:** Schedule of measurements.

Variable	Instrument	Time Point
*Self-report*		
Premenstrual symptoms	DRSP	Two cycle baseline, each treatment cycle, natural follow up
Psychiatric status (mood disorders only)	Structured Clinical Interview (for DSM-5)-Research Version (SCID-1/RV) *	Screening
Depression, anxiety, stress	Depression and Anxiety Stress Scales-42 (DASS-42)	Baseline, each treatment cycle
Quality of life, wellbeing	Women’s Quality of Life Questionnaire (WOMQOL)	Baseline, each treatment cycle
Sleep quality	Pittsburgh Sleep Quality Index (PSQI)	Baseline, each treatment cycle
Contextual stress	The Perceived Stress Scale (PSS)	Baseline, each treatment cycle
Sexual satisfaction	The New Sexual Satisfaction Scale-Short From (NSSS-S)	Baseline, each treatment cycle
Diet quality, alcohol and drug intake	Drug, Alcohol, and Food Questionnaire (DAF)	Baseline, each treatment cycle
Treatment side effects	Side Effects Questionnaire	Baseline, each treatment cycle
Treatment efficacy	Clinical Global Impression scale (CGI scale, Improvement scale)	End of treatment
*Observer report*		
Change in premenstrual symptoms	Modified DRSP	Baseline, end of treatment

* Clinician administered.
